# Direct measurement of Coulomb-laser coupling

**DOI:** 10.1038/s41598-020-79805-x

**Published:** 2021-01-12

**Authors:** Doron Azoury, Michael Krüger, Barry D. Bruner, Olga Smirnova, Nirit Dudovich

**Affiliations:** 1 Department of Physics of Complex Systems, Weizmann Institute of Science, Rehovot, 76100 Israel; 2 Department of Physics, Massachusetts Institute of Technology, Cambridge, MA 02139 USA; 3 Department of Physics and Solid State Institute, Technion—Israel Institute of Technology, Haifa, 32000 Israel; 4 Max-Born-Institut, Max-Born-Straße 2A, 12489 Berlin, Germany; 5 Technische Universität Berlin, Ernst-Ruska-Gebäude, Hardenbergstraße 36A, 10623 Berlin, Germany

**Keywords:** Attosecond science, High-harmonic generation

## Abstract

The Coulomb interaction between a photoelectron and its parent ion plays an important role in a large range of light-matter interactions. In this paper we obtain a direct insight into the Coulomb interaction and resolve, for the first time, the phase accumulated by the laser-driven electron as it interacts with the Coulomb potential. Applying extreme-ultraviolet interferometry enables us to resolve this phase with attosecond precision over a large energy range. Our findings identify a strong laser-Coulomb coupling, going beyond the standard recollision picture within the strong-field framework. Transformation of the results to the time domain reveals Coulomb-induced delays of the electrons along their trajectories, which vary by tens of attoseconds with the laser field intensity.

## Introduction

Strong-field light-matter interactions initiate a wide range of fundamental phenomena—from high harmonic generation (HHG)^1^ to photoelectron holography^2^ or laser induced diffraction^3^. At an early stage of this field of research, light-matter phenomena were described via their interaction with strong laser field only, while all additional forces have been neglected. However, over the past two decades the important role of the Coulomb potential has been revealed. Notable examples include Coulomb enhanced emission at low kinetic energies of strong-field photoionization^4–6^, Coulomb focusing^7,8^, which amplifies the yield of HHG, or nonsequential double ionization^9^. Recently, it was shown that the Coulomb interaction plays an important role in the interpretation of attoclock experiments^10–12^. These experiments share one common property—the Coulomb effect is estimated via its influence on the experimental observable such as momentum distribution or ionization yield. However, the Coulomb interaction influences the entire complex electron wavefunction, both its amplitude as well as its phase. As in many phenomena in nature, the phase information encodes valuable spatio-temporal properties of the interaction, which are hidden in intensity measurements. Resolving this phase requires an interferometric measurement that probes the electron as it interacts with the combined laser-Coulomb potential, on an attosecond time scale.

**Figure 1:**
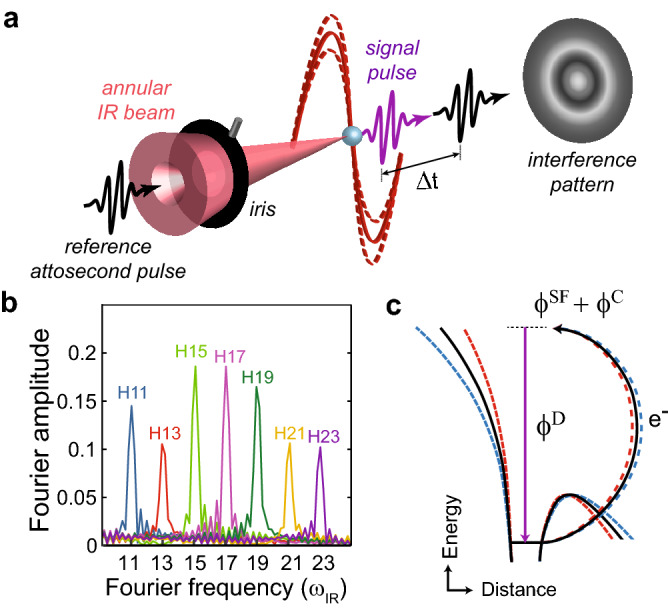
Differential phase measurement using XUV-XUV interferometry. **(a)** Experimental scheme. A delay controlled APT (black) and its generating annular IR pulse (red) are focused into a gas target, where a second phase-locked and independent APT (purple) is generated by the same IR pulse. The interference signal between the two APTs as a function of delay is spectrally resolved and recorded in the far-field for a range of IR intensities, controlled by a motorized iris. **(b)** Fourier amplitudes of an interference signal extracted from a delay scan where argon is used in both sources. Harmonics 11 to 23 oscillate at their fundamental frequency. **(c)** Schematic description of the phase accumulated during the HHG process. The phase of the emitted XUV photon encodes all the steps of the HHG interaction: strong-field tunneling and acceleration $\phi ^{\rm {SF}}$ and the dipole transition phase $\phi ^{\rm {D}}$ at recollision. In addition, the electron accumulates phase $\phi ^{\rm {C}}$ as it interacts with the ionic Coulomb potential. Changing the IR intensity leads to a variation of the electron trajectory, as indicated by the red and blue lines for higher and lower intensity, respectively. As a result, both $\phi ^{\rm {SF}}$ and $\phi ^{\rm {C}}$ are modified, while $\phi ^{\rm {D}}$ remains unchanged.

The phase associated with the Coulomb interaction is naturally encoded in the HHG process. In HHG, a strong laser field liberates a bound electron by tunneling ionization, and drives it back to the ionic core, where it recollides, leading to the emission of an high-energy photon in the extreme ultraviolet (XUV) range^13,14^. The phase of the emitted radiation is dictated by all steps of the interaction (see the schematic description in Fig. 1c). Importantly, this phase probes the interaction of the accelerated electron with the ionic Coulomb potential. However, since the emitted HHG radiation couples all steps of the interaction, isolating and resolving the Coulomb-induced phase is highly challenging.

In this paper we obtain a direct insight into the Coulomb interaction and resolve the phase accumulated by the laser-driven electron as it interacts with the Coulomb potential. Applying advanced XUV-XUV interferometry^15,16^, we isolate the Coulomb-induced phase with attosecond precision over a large spectral range. This scheme is based on a complete and independent control over two phase-locked HHG sources, and the ability to scan their relative delay with attosecond precision. By manipulating the strong-field intensity at one of the HHG sources, we obtain an interferometric study of the Coulomb phase scaling with the light intensity. Our measurements reveal, for the first time, the appearance of laser-Coulomb coupling, which goes beyond the standard description of HHG in the framework of the strong-field approximation (SFA^17–20^). Transferring the phase measurements into the time-domain resolves Coulomb-induced delays, by tens of attoseconds, accumulated by the free electron as it recollides with the parent ion.

## Results

### Interferometric detection of laser-Coulomb coupling

We resolve the laser-induced Coulomb phase by interfering a reference HHG source, which remains constant throughout the experiment, with a target HHG source, that is generated with different infrared (IR) intensities. In the experimental setup (for detailed description see Methods), a 25-fs, 794 nm laser pulse is focused into a gas cell generating a reference attosecond pulse train (APT) (black pulse in Fig. 1a). A thin Al foil transmits the APT while the inner part of the IR beam is blocked. Both beams co-propagate and are refocused into a second gas source by a curved two-segment mirror where the remaining annular part of the IR beam is independently generating a target APT (purple pulse in Fig. 1a). The intensity of the IR beam at the target source can be independently tuned by a motorized iris, placed after the two-segment mirror. The temporal delay $\Delta t$ between the reference and the target APTs is controlled by moving the inner segment of the concave focusing mirror. An XUV spectrograph resolves the interference of the two APT beams.

In our experiment we vary the intensity at the target source, while all other experimental conditions such as the gas medium and pressure remain unchanged. Scanning $\Delta t$ records the interference signal of the two sources, where the intensity of each harmonic number oscillates at its own fundamental frequency (Fig. 1b). Performing a Fourier analysis enables us to extract both the amplitude as well as the phase of the interference signal. Note that this approach is conceptually different from well established interferometric techniques based on photo-electron spectroscopy, such as reconstruction of attosecond beating by interference of two-photon transitions (RABBITT) and streaking^21–23^. While these methods can be used to measure the derivative of the phase with respect to energy, the absolute phase of each frequency component remains inaccessible. Furthermore, the RABBITT technique is limited to weak IR fields, whereas our experiment allows a large dynamic range of the IR intensity.

**Figure 2:**
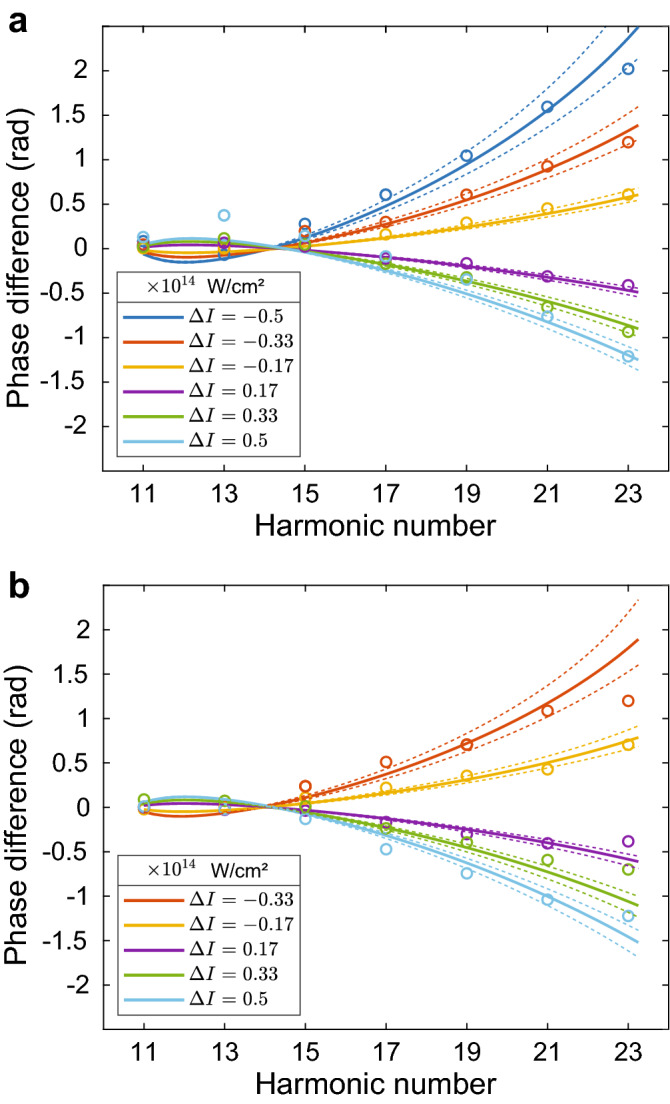
Differential phase measurements of the XUV phase. **(a,b)** Measured (circles) and calculated (lines) $\Delta \phi (\Omega ,\Delta I)$ for argon **(a)** and nitrogen **(b)** at harmonics 11–23. For both targets, the reference intensity $I_0$ is $1.46\times 10^{14}\,{\rm {W\,cm}}^{-2}$. The dashed lines represent the uncertainty in the IR intensity.

We perform a systematic study of the Coulomb phase by recording the interference signal for different intensity levels $I_j$ at the target source, where we define $I_0$ as the median intensity. The Fourier phase $\phi (\Omega ,I_j)$ represents the relative spectral phase difference between the reference source, $\phi _{\rm {ref}}(\Omega )$, and the target source, $\phi _{\rm {tar}}(\Omega ,I_j)$, where $\Omega$ is the harmonic frequency. We repeat the delay scan with different intensities at the target source while keeping $\phi _{\rm {ref}}(\Omega )$ constant. Therefore, extracting the phase differences $\phi (\Omega ,I_j)-\phi (\Omega ,I_0)$ cancels out $\phi _{\rm {ref}}(\Omega )$. Accordingly, we are left with the absolute phase shifts at the target source itself, $\Delta \phi (\Omega ,\Delta I)=\phi _{\rm {tar}}(\Omega ,I_0+\Delta I)-\phi _{\rm {tar}}(\Omega ,I_0)$, as a function of the intensity difference $\Delta I = I_j - I_0$. Finally, we control the intensity by modifying the beam diameter, therefore $\Delta \phi (\Omega ,\Delta I)$ includes an additional contribution from the beam diameter dependent Gouy phase. We isolated and removed the Gouy phase by repeating the same set of differential phase measurements without the Al filter, allowing an interferometric measurement at the fundamental IR frequency $\omega _{\rm {IR}}$. Accordingly, we resolve the Gouy phase shift for each harmonic number by extracting the Fourier phase at $\omega _{\rm {IR}}$ (see Methods for more information). Figure 2a,b show the measured (circles) and calculated (lines) $\Delta \phi (\Omega ,\Delta I)$, for argon or molecular nitrogen as the target gas, respectively.

### Laser-Coulomb phase calculations

To analyze our results we theoretically model the spectral phase extracted by the interferometric measurement. Figure 1c provides a schematic description of the HHG mechanism, illustrating the main contributions to the harmonic phase. This picture arises due to the semiclassical approximation for the electron dynamics in a strong laser field. This well established approximation describes the electron dynamics between ionization and recombination in terms of a set of classical trajectories. Each harmonic is associated with a single trajectory (here we consider only short trajectories, see^24^) which is characterized by the ionization time $t_{\rm {i}}(\Omega )$, the time the electron appears at the continuum, and the recombination time $t_{\rm {r}}(\Omega )$, the time it recombines with the core. Following this picture, the phase of the emitted harmonics can be factorized into three contributions: the strong-field tunneling and propagation phase $\phi ^{\rm {SF}}$, the dipole photo-recombination phase $\phi ^{\rm {D}}$ and the phase $\phi ^{\rm {C}}$ accumulated due to electron interaction with the Coulomb potential of the core. Accordingly, the differential phase measurement is described as:

1 $$\begin{aligned} \Delta \phi (\Omega ,\Delta I)=\Delta \phi ^{\rm {SF}}(\Omega ,\Delta I)+\Delta \phi ^{\rm {C}}(\Omega ,\Delta I)+\Delta \phi ^{\rm {D}}(\Omega ). \end{aligned}$$

Such factorization leads to intensity independent $\phi ^{\rm {D}}$ and therefore the last term should cancel out in our measurement. The strong-field contribution $\Delta \phi ^{\rm {SF}}(\Omega ,\Delta I)$ can be captured quantitatively by the well-established SFA^15,20,25,26^. Following the SFA, $\phi ^{\rm {SF}}$ can be expressed in atomic units as:

2 $$\begin{aligned} \phi ^{\rm {SF}}(\Omega ) = {\rm {Re}} \left\{ {\Omega t_{\rm {r}}(\Omega ) - \int _{t_{\rm {i}}(\Omega )}^{t_{\rm {r}}(\Omega )} \left( \frac{[v_r + A(t)-A(t_{\rm {r}})]^2}{2} + I_{\rm {p}} \right) dt} \right\} , \end{aligned}$$

where $v_r$ is the electron velocity at the time of recombination. *A*(*t*) is the time-dependent vector potential of the infrared laser field and $I_{\rm {p}}$ the ionization potential of the atom.

The calculation of the Coulomb phase $\phi ^{\rm {C}}$ is challenging due to the intrinsic singularity at the ionic core. This challenge can be addressed by a boundary-matching procedure within the analytical R-matrix (ARM) approach^27^, or by the complex-valued quantum orbit approach^28^. Here we use the ARM approach for calculating the Coulomb phase as a function of IR intensity. The Coulomb phase shift is given by

3 $$\begin{aligned} \Delta \phi ^{\rm {C}} (\Omega ) = - {\rm {Re}} \int _{t_\kappa }^{t_{\rm {end}}} V [r(t)] dt, \end{aligned}$$

where *V*(*r*) is the Coulomb potential and *r*(*t*) is the classical trajectory describing the motion of free electron in the laser field. The boundaries $t_\kappa$ and $t_{\rm {end}}$ are chosen in order to avoid the Coulomb singularity (see^27^ for more details).

**Figure 3:**
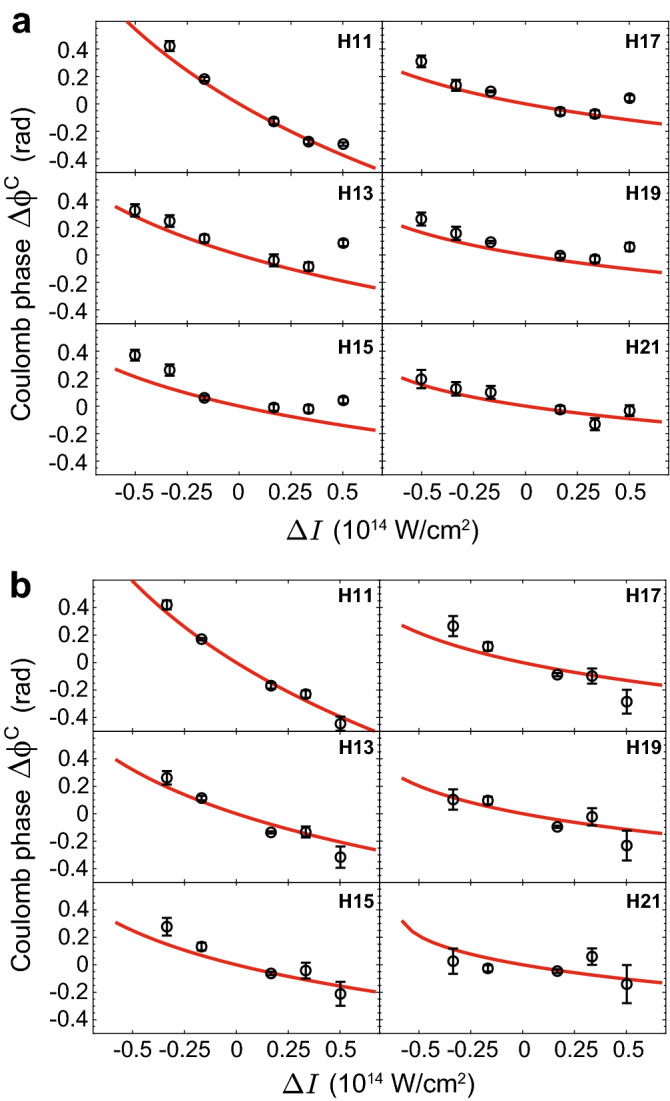
Differential phase measurements of the Coulomb phase. **(a,b)** Measured (black symbols) and calculated (red lines) Coulomb phase as a function of intensity variation in argon **(a)** and nitrogen **(b)**. In both the measurements and the calculations, we subtract the strong-field phase from the phases presented in Fig. 2a,b.

Next, we extract the quantity of interest—the Coulomb phase in the HHG process as a function of the IR intensity. We isolate the Coulomb phase by subtracting the calculated SFA phase from the measured phase differences (see Fig. 3a,b for argon and molecular nitrogen, respectively). We find an excellent agreement between the measured (black symbols) and the calculated (red lines) Coulomb phase shifts over a large energy range that spans between harmonics 11 and 21.

## Discussion

Two main observations can be made from our experimental results, for both argon and nitrogen. First, we find that the Coulomb phase shift decreases with increasing intensity, for all harmonics. Second, we identify that the Coulomb phase modification with intensity increases as the harmonic number is reduced and approaches the threshold harmonic, harmonic 11. What is the origin of this dependence and the insight it provides?

The phase accumulated by the electron as it interacts with the laser field changes with the trajectory length. As we increase the laser intensity, the trajectory length—and therefore the phase associated with each harmonic order—decreases. Indeed, our experimental results capture this descending trend. The dependence of the magnitude of the Coulomb phase shift on intensity is dictated by the acceleration of the electron. As we modify the laser intensity we change the time the electron spends at the core vicinity, and therefore its acceleration towards the recollision velocity $v_{\rm {r}}(\Omega ) = \sqrt{2(\Omega - I_{\rm {p}})}$, which reduces with the harmonic number. For the slow returning electrons, an intensity modification will have a larger effect on the time it travels close to the ionic core. Therefore, the Coulomb phase shifts at the energy range close to the ionization threshold will be larger compared with higher harmonics. We emphasize that for all harmonics $v_{\rm {r}}(\Omega )$ itself does not depend on intensity, rather, it is the acceleration of the electron before recollision which varies with intensity.

**Figure 4:**
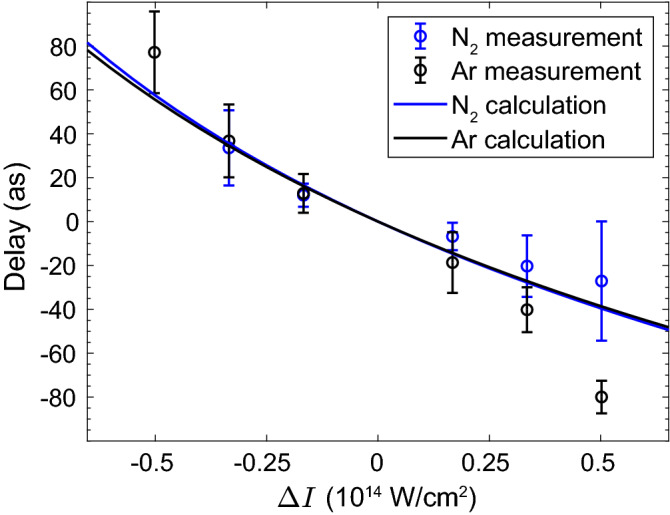
Coulomb time delays. Measured (circles) and calculated (lines) Coulomb induced delays as a function of intensity variation, extracted for electrons with a final kinetic energy of approximately 3 eV, in argon (black) and nitrogen (blue).

A deeper insight into the dynamical properties of the Coulomb phase shifts can be obtained by calculating the time delay associated with our experimental results. Following^27^, we can calculate a Coulomb time delay $t^{\rm {C}}$ which characterizes the temporal shift of the recollision time due to the long-range Coulomb interaction, compared to a short-range potential, namely the SFA recollision time. This time delay is given by:

4 $$\begin{aligned} t^{\rm {C}}(\Omega ) = - \frac{\partial \phi ^{\rm {C}}(\Omega )}{\partial \Omega }. \end{aligned}$$

Equation (4) resembles the form of the well-known Wigner–Smith photoionization time delay^29,30^. In our experiment we measure the scaling of $\phi ^{\rm {C}}$ with intensity, therefore we can extract the intensity-dependent Coulomb time delay $\Delta t^{\rm {C}} (\Omega , \Delta I) = \Delta t^{\rm {C}} (\Omega ,I_0 +\Delta I) - \Delta t^{\rm {C}} (\Omega ,I_0)$. We retrieve $\Delta t^{\rm {C}}$ from our experimental data by taking the phase differences between pairs of neighboring harmonics,

5 $$\begin{aligned} \Delta t (N\omega _{\rm {IR}}, \Delta I) \approx - \frac{\phi ^{\rm {C}}((N+1)\omega _{\rm {IR}}) - \phi ^{\rm {C}}((N-1)\omega _{\rm {IR}})}{2 \omega _{\rm {IR}}}, \end{aligned}$$

where *N* is the harmonic order. Figure 4 shows the resulting curve for the time delay extracted for $N = 12$, which corresponds to a final kinetic energy of approximately 3 eV, for both Ar and nitrogen. Both the experimental as well as theoretical delay spans between − 50 and 80 attoseconds, and decreases with the laser intensity. At the higher intensity range we find deviations between the measured and calculated delays.

In the tunneling limit, we can express the Coulomb delay as $t^{\rm {C}}(\Omega )\propto E(t_{\rm {r}}) v_{\rm {r}}^{-3}$, where $E(t_{\rm {r}})$ is the electric field strength at the time of recollision, calculated using the SFA^27,31^. This expression provides an intuitive physical picture to the delays resolved in our experiment. For a given return velocity, mapped to each harmonic number, increasing the driving field intensity results in a decrease of both $t_{\rm {r}}$ and $E(t_{\rm {r}})$, leading to the decrease of the delays as identified in Fig. 4. In addition, as we approach the threshold harmonics, both the return velocities as well as the instantaneous field at $t_{\rm {r}}$ are reduced, inducing significant delays.

In conclusion, we have isolated and measured, for the first time, the accumulated Coulomb phase within the HHG interaction by applying an interferometric measurement scheme. Our measurement reveals the strong coupling of the Coulomb potential with the laser field. Such coupling increases at harmonics which are closest to the ionization threshold, due to the low momenta of the recolliding electrons associated with these harmonics. Finally, transformation of the phase measurement to the time domain reveals intensity-dependent Wigner-like delays for electrons with low final kinetic energy.

Looking forward, we believe that the work presented here will stimulate further experimental and theoretical studies, aiming at the measurement of ultrafast electron dynamics in a wide variety of atomic and molecular systems via XUV interferometry. An intriguing example is time delays of electron dynamics due to auto-ionization resonances, a multi-electron phenomenon which could be studied so far only in the weak field regime^32–35^. The results presented in this work will open the door for extending this study to the strong-field regime. It will enable the measurement of strong-field-driven modifications of ionization and recombination dipoles or atomic and molecular resonances—all imprinted on the phase of the emitted harmonics field.

## Methods

### Experimental setup and measurement scheme

**Figure 5:**
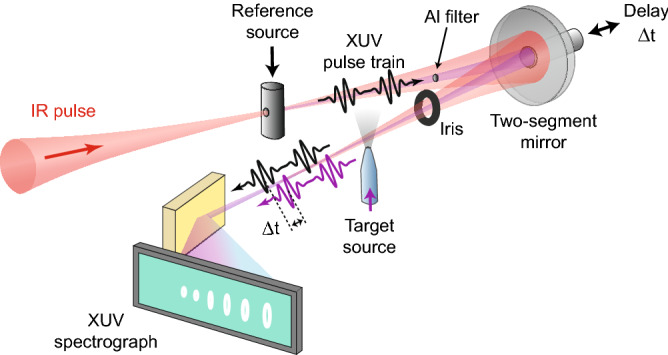
Schematic description of the XUV–XUV interferometer. For a detailed description see the text.

Figure 5 shows a schematic sketch of the experimental setup for collinear XUV-XUV interferometry of two independent sources. An amplified Ti:sapphire laser system operated at 1 kHz repetition rate delivers $\sim$ 23 fs pulses at a central wavelength of 792 nm. Focusing the beam into a continuous flow gas cell filled with argon generates the reference APT. We spatially separate the co-propagating IR and APT beams by a thin aluminum filter (200 nm thickness). Both beams are then refocused by a curved two-segment mirror (750 mm focal length) into the target gas (continuous flow glass nozzle, orifice of approximately 10 $\upmu$m) in order to produce the target APT which interferes with the reference APT. The position of the target source with respect to the IR focus is adjusted to produce short trajectories of HHG. The inner part of the focusing mirror reflects the APT in the spectral range of 17–51 eV. A piezo stage controls the temporal delay $\Delta t$ between the reference APT and target APT with a step size of 6.7 as and an accuracy of about 1 as. The IR intensity at the target gas can be adjusted independently by means of a motorized iris. The co-propagating APT beams are spectrally resolved by a flat-field aberration-corrected concave grating and recorded by a micro-channel plate detector, imaged by a CCD camera. We performed the experiment for argon or molecular nitrogen in the target gas. The target’s local gas pressure was kept at a low value of 1.3 Torr throughout the experiment in order to suppress undesired correlations between the reference APT and the properties of target gas medium. Such a correlation can be caused by the strong ionization of the target gas by the IR pulse, leading to the presence of electron plasma during the interaction. A delay-dependent phase shift of the reference APT may result. A conservative estimate of this shift in our experiment (1.3 Torr, room temperature, full ionization, propagation length of 100 $\upmu$m) yields an upper bound of $10^{-3}$ rad, much smaller than the experimental error bars. We therefore neglect this effect.

In each scan, we varied $\Delta t$ over a range of 6.7 fs (about 2.5 IR cycles) and recorded the XUV spectrum. We applied the differential scheme by repeating the delay scan multiple times, changing the IR intensity at the target source for every other scan. Importantly, we repeat the measurement for $I_0$ after every measurement of higher or lower intensity, providing eight independent measurements for each intensity variation. We overcome the macroscopic averaging due to phase matching effects by performing a spatial analysis of the HHG signal at the spectrograph. For each harmonic we selected the detector pixel that exhibits the most significant signal-to-noise ratio, located in the region where the short-trajectory HHG signal dominates. For each scan, we extracted the Fourier phase $\phi (\Omega )$ as a function of harmonic number. Note that the Fourier analysis shows no trace of coupling between the reference APT and the IR pulse in the target gas medium^15^. Based on the measured phases, we calculate the experimental phase differences as a function of intensity variation $\Delta \phi (\Omega ,\Delta I)$. Finally, we averaged over the eight pairs of phase differences in each harmonic and determined the error of the mean of the phase differences. In order to estimate the slow thermal drift of the piezo (up to 50 as per hour), we determined the temporal drift between pairs of identical measurements of every target source. We corrected for the drift, resulting in a systematic error in the experimental group delay (or equivalently, a linear phase shift). Typically, this error amounts to about 5 as, translating into a frequency-dependent error in the phase differences of $N \cdot 0.012$ rad (*N*: harmonic number).

### Gouy phase measurement

Controlling the IR intensity at the target source by changing the beam diameter introduces an additional geometrical phase shift into the differential phase measurement scheme. In this section we describe how we directly measure this phase shift, and subsequently remove it from our experimental results. The phase of a focused beam is shifted by $\pi$ when moving through the focal plane. This fundamental optical phenomenon, known as the Gouy phase shift, can be formulated for a gaussian beam as $\phi ^{\rm {(G)}}=\arctan ( Z/Z_{\rm {R}})$, where *Z* denotes position along the propagation direction. The Gouy phase shift strongly depends on the Rayleigh range $Z_{\rm {R}}=\pi W_0^2 /\lambda$, where $W_0$ and $\lambda$ are the beam waist and wavelength, respectively. In our experiment, changing the diameter of the motorized iris (see Fig. 5) leads to modifications of the focal Rayleigh range. Accordingly, for a given position of the tip of the gas nozzle within the Rayleigh range, the Gouy phase of the IR beam that drives HHG at the nozzle position will vary with iris size. A phase shift of the IR beam translates into a linear phase shift of the generated harmonics according to $\phi ^{\rm {(G)}}(\Omega )=N \phi ^{\rm {(G)}}(\omega _{\rm {IR}})$, where *N* is the harmonic number. Therefore the differential phase measurements (Equation 1 in the main text) include an additional phase shift $\Delta \phi ^{\rm {(G)}}(\Omega ,\Delta I)=N [ \phi ^{\rm {(G)}}(\omega _{\rm {IR}},I_0 +\Delta I)-\phi ^{\rm {(G)}}(\omega _{\rm {IR}},I_0)]$.

**Figure 6:**
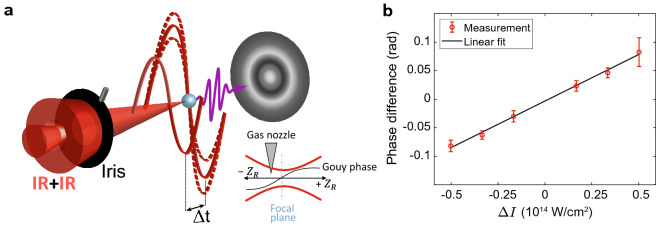
Measurement of the Gouy phase variation. **(a)** Experimental scheme. By turning off the reference HHG source and removing the Al filter, we induce an interference between the inner and outer parts of the IR beam. Scanning the relative delay leads to oscillations of the HHG signal at the target gas nozzle with the IR frequency $\omega _{\rm {IR}}$. Varying the iris affects only the outer part of the IR beam, which enables us to determine the Gouy phase shift as a function of iris size from the Fourier analysis of the HHG signal oscillations. **(b)** Experimental Gouy phase variation as a function of the intensity variation $\Delta I$. The black line represents a linear fit to the experimental data (red points).

In order to measure the Gouy phase shift and remove it from the intensity-dependent phase measurement results, we performed an additional interferometric measurement (see Fig. 6a). We turned off the reference APT and removed the Al filter such that the inner and outer parts of the IR beam can interfere at the target source. Importantly, since the diameter of the inner part of the IR beam is smaller than the minimal diameter of the iris, it is not affected by the iris variation, providing a constant Gouy phase reference. We repeated exactly the same set of differential phase measurements as described in the main text for argon and nitrogen, however in this case the intensity of all harmonics oscillate at $\omega _{\rm {IR}}$. Since the phase is measured through the HHG signal and the nozzle position is fixed for all measurements we can link the measured phase to the variation of the Gouy phase shift at the position of the HHG interaction region $\Delta \phi ^{\rm {(G)}}(\Omega ,\Delta I)$. Figure 6b shows the measured Gouy phase shifts as a function of IR intensity variation at the target source. In order to reduce the IR pulse envelope averaging effects, the phase was extracted from a Fourier analysis over the highest harmonics 21 and 23. Finally, we applied a linear fit to the measured phases, and used it for removing the Gouy phase shifts from the results in the main text according to $\Delta \phi ^{\rm {(G)}}(\Omega ,\Delta I)$. Note that since the Gouy effect is global, we use a single correction for all measurements, for both argon and nitrogen experiments, and find excellent agreement with the SFA and Coulomb phase calculations (Figure 2 in the main text).

## Data Availability

The data that support the findings of this study are available from the corresponding author upon reasonable request.
